# Toward a Psychology of Ideas Rather Than Demographics: Commentary on Hommel (2024)

**DOI:** 10.1177/17456916241236167

**Published:** 2024-04-23

**Authors:** Keith E. Stanovich

**Affiliations:** Department of Applied Psychology and Human Development, University of Toronto

**Keywords:** identity politics, quotas, ideological bias

## Abstract

The public will rightly not value a science that is more concerned with demographic population matching than with ideas. Taking further steps in the direction of identity politics will reduce public confidence in psychology’s conclusions and reduce trust and respect. If psychology embraces demographic quotas, there will be self-selection out of the discipline, and that self-selection will harm our science.

**Publisher’s Note**

This article is part of a collection of articles related to the December 2022 APS Vote of No Confidence in the Editor-in-Chief of *Perspectives on Psychological Science*. Please see the editorial [https://doi.org/10.1177/17456916241246556] in this issue for further details on the collection.

Recently there have been many discussions about ideological bias in psychology impeding necessary scientific checks and balances (Campbell & Manning, 2018; Ceci & Williams, 2018; Crawford & Jussim, 2018; Duarte et al., 2015; Jussim, 2018, 2022). Roberts et al. (2020) and Hommel (this issue) focus on the issue of race as a factor in the intellectual composition of psychology. I have written on ideological bias (Stanovich, 2021) but not race and see some analogies and disanalogies between the two that are worthy of comment.

Political ideology is a direct indicator of differences in worldview that may impact the way that many topics in psychology are studied (educational achievement gaps, family structure, poverty, violence, parenting, sexual orientation, crime, immigration, environmentalism, drug addiction, fairness, etc.). In contrast, race is a very indirect indicator of perspective variability because it is only moderately related to differences in worldview.

Ratios of 10 liberals to 1 conservative are common in psychology, whereas the population ratio is closer to 1:1 (Buss & von Hippel, 2018; Duarte et al., 2015; Jussim, 2022). However, a well-trained psychologist would be the first to point out that even discrepancies this large do not automatically mean that bias or discrimination is at work. Gross (2013) explored the many alternative hypotheses for the ideological disparity in academia and concluded that self-selection was the dominant factor, and it was a much more important contributor than bias or discrimination (but see Inbar & Lammers, 2012; Jussim, 2021, 2022).

It should be noted that the analysis by Gross (2013) occurred prior to the advent of DEI statements as a criterion for employment in many universities. Such statements now function like ideological loyalty oaths (McBrayer, 2022), screening out anyone who cannot convincingly support current progressive positions regarding race and gender (Huemer, 2022; Jussim, 2019; Rozado, 2019; Small, 2021; Thompson, 2019). As the report of Foundation for Individual Rights and Expression (2022) on DEI statements points out, these statements require applicants to take a specific position on social and political issues that are highly contested. As DEI statements become even more prevalent, they will become an overtly discriminating mechanism against conservative applicants (or indeed even liberal Democrats who balk at endorsing the specific terms and positions of identity politics).

Unlike the case of DEI statements which can prevent the hiring of conservative faculty, there are no overt barriers to hiring racial minorities on psychology faculties. Indeed, the implementation of affirmative action and “diversity hires” at the faculty level signal just the opposite. Overt attempts to include minorities are common across the academic landscape. If indeed an underrepresentation does exist, it is certainly due to a pipeline effect (Gross, 2013; Kuncel & Worrell, 2022; Woessner & Kelly-Woessner, 2009)—starting long before the faculty hiring process commences. Attempts to prove bias and discrimination in research psychology thus must move the focus away from standard analyses of hiring practices, and this is just what Roberts et al. (2020) do. They choose a very curious way to operationalize bias and “structural inequality” in psychology—by examining the number of articles published in three areas of psychology that “highlight the role of race in human psychology.” This is an odd metric because there is no way of determining the *absolute* number of such articles needed to indicate a completely unbiased psychology. What is the number of concepts/categories that could possibly be highlighted in psychology journal articles? The population space here is totally unknown. There is no way to determine how many articles in psychology should highlight religion, or highlight social class, or highlight sex, for example. Furthermore, there is no logical or empirical model that dictates that the intellectual category of “number of articles that highlight race” has any relation at all to the number of people of color in the population—no more so than the number of articles that highlight religion should have any relationship to the number of people in the population who are non-atheists.

Hommel (this issue) implies that Roberts et al. (2020) are committing a version of the disparity fallacy (a termed coined by Hughes, 2018), and indeed this seems to be the case. The disparity fallacy is the idea that any difference in an outcome variable that is viewed as unfavorable to a minority group must be due to discrimination (Clark & Winegard, 2020; Goldberg, 2021; Sowell, 2019). Of course, all psychologists know that discrimination is only one of many possible alternative explanations for any group difference. And they also know that eliminating alternative explanations is the way to get at the proper causal model for the disparity. Psychologists should also be prominent in explaining to the public that remedies for social problems depend upon having the right causal model.

As mentioned previously, the research by Gross (2013) indicated that even a disparity as massive as the 10 to 1 ideological difference in social-science faculties is not necessarily due to discrimination. In that case, an alternative hypothesis (self-selection) seemed to be more determinative than the hypothesis of bias. Disparity alone is not an indication of bias or discrimination, contrary to the assumptions of many current anti-racism programs. Indeed, the *ideological* imbalance in psychology probably accounts for the discipline’s embarrassing silence regarding the falsity of the group disparities model that lies behind many current anti-racism efforts (“As an anti-racist, when I see racial disparities, I see racism,” Kendi, 2018; “We have a hard time recognizing that racial discrimination is the sole cause of racial disparities in this country and in the world at large,” Kendi, 2016, p. 10).

Hommel (this issue) probably sees the disparity fallacy in Roberts et al. (2020) because of words like “structural inequality” and “systemic inequality” in the latter. Hommel’s conjecture is reinforced by a media report of this research titled “Psychological Research Has a Racism Problem, Stanford Scholar Says” (deWitte, 2020), and by the fact that the authors seem to endorse a quota-based solution: “This means that journals should consist of diverse editors, reviewers, authors, and participants—*ideally at rates that mirror diversity at the national level or within psychology* [emphasis added]” (p. 1304). I deliberately employ the “Q-word” (quota) here. Those of us who have been in academia for a while know that the genteel euphemism “diversity” will be used to usher in an industrial-sized bureaucracy of bean counters to “establish a diversity task force” (p. 1304) to “ensure that the recommendations are monitored and enacted” (p. 1304)—thus, quotas.

Roberts et al. (2020) acknowledge that their analysis and recommendations generalize: “The core issues tackled here extend to other social groups as well, including but not limited to those based on gender, sexual orientation, religion, class, and political orientation (p. 1304).” Hommel worries about the combinatorial explosion of demographic and psychological categories this entails.

I too worry about the implications of all these recommendations, particularly as they are filtered through an intrusive university and research bureaucracy that drains more faculty energy every day. What happens when “ideally at rates that mirror diversity at the national level or within psychology” is “extended to other social groups as well” and then a “diversity task force . . . ensures that the recommendations are monitored and enacted.” The psychological research community will become more like what parts of the university are now—focused more on progressive credentials than on advancing knowledge. We want contending ideas in psychology, not contending cells in a 3 × 3 × 2 × 6 × whatever matrix of demographic characteristics, especially if (as discussed in the context of race above) the categories in question are not a direct measure of diverse thinking but only indirect indicators of probabilistic differences.

No one who decries the intellectual monoculture within universities wants to see a quota system that mandates the hiring of equal percentages across the ideological spectrum. Likewise, we do not want a science built around demographic quotas. Such mandatory population matching will undermine the field and reduce its status and impact on public policy. The public will (rightly) not value a science that operates on that basis. Population quotas will end up undermining our science just like affirmative action undermines the status of high-achieving minorities in college admissions—by casting doubt on the qualifications of all minority candidates (Loury & Sandel, 2020). Applying quotas to our science will likewise reduce public confidence in its conclusions and reduce trust and respect. A quota-based science will end up being a Pyrrhic victory for advocates of identity politics. They will inherit a psychology that the public does not believe or respect.

The use of quotas will also cause an exodus from our discipline among those who value ideas over demographics. Potential psychologists are already choosing to conduct their work from within other disciplines such as economics, linguistics, neuroscience, business administration, marketing, computer science, and human factors engineering. Others are doing their work in think tanks and independent institutes outside of academia altogether. If psychology lurches in the direction of demographic quotas, there will be more self-selection out of the discipline, and that self-selection will harm our science.

## Transparency

*Action Editor:* Klaus Fiedler

*Editor:* Klaus Fiedler
